# Phlegmasia Dolens, Gonorrhœa, and Rheumatism

**Published:** 1845-04

**Authors:** 


*Phlegmasia Dolens, Gonorrhœa, and Rheumatism.* — Mr. Hancock detailed the following case: A man came under his care at the hospital with gonorrhœa, which had existed six or eight weeks; the discharge ceased, and the man was seized with gonorrhœal inflammation of the knee-joint; this spread to the ankle, and the wrist-joint. One morning he complained of great pain in one of the legs, and on making examination, it was found to be affected with complete phlegmasia dolens, and the femoral vein was evidently hardened. On examining the penis, the veins on its dorsum was hard as a cord, and thickened. Now might not the inflammation of the veins of the penis have spread to the pelvic veins, and afterwards to the veins of the leg? Phlegmasia dolens appeared to arise from inflammation of the uterine veins, and there seemed, throughout his case, to be something analogous, for in patients dying of inflammation of the veins, pus was often found in the joints. The patient, in this case, recovered under antiphlogistic treatment. He (Mr. Hancock) had seen cases of gonorrhœa in which, when the discharge suddenly ceased, there would be pleuritis, or inflammation of the joints. He had seen one case in which peritonitis came on from this cause.—*Ib.*

